# Hot dense silica glass with ultrahigh elastic moduli

**DOI:** 10.1038/s41598-022-18062-6

**Published:** 2022-08-17

**Authors:** Ningyu Sun, Zhu Mao, Xinyue Zhang, Sergey N. Tkachev, Jung-Fu Lin

**Affiliations:** 1 Laboratory of Seismology and Physics of Earth’s Interior, School of Earth and Space Sciences, University of Science and Technology of China, Hefei, Anhui 230026 China; 2 CAS Center for Excellence in Comparative Planetology, University of Science and Technology of China, Hefei, Anhui 230026 China; 3 Frontiers Science Center for Planetary Exploration and Emerging Technologies, University of Science and Technology of China, Hefei, Anhui 230026 China; 4 Center for Advanced Radiation Sources, University of Chicago, Chicago, IL 60637 USA; 5 Department of Geological Sciences, Jackson School of Geosciences, The University of Texas at Austin, Austin, TX 78712 USA

**Keywords:** Mineralogy, Condensed-matter physics

## Abstract

Silicate and oxide glasses are often chemically doped with a variety of cations to tune for desirable properties in technological applications, but their performances are often limited by relatively lower mechanical and elastic properties. Finding a new route to synthesize silica-based glasses with high elastic and mechanical properties needs to be explored. Here, we report a dense SiO_2_-glass with ultra-high elastic moduli using sound velocity measurements by Brillouin scattering up to 72 GPa at 300 K. High-temperature measurements were performed up to 63 GPa at 750 K and 59 GPa at 1000 K. Compared to compression at 300 K, elevated temperature helps compressed SiO_2_-glass effectively overcome the kinetic barrier to undergo permanent densification with enhanced coordination number and connectivity. This hot compressed SiO_2_-glass exhibits a substantially high bulk modulus of 361–429 GPa which is at least 2–3 times greater than the metallic, oxide, and silicate glasses at ambient conditions. Its Poisson’s ratio, an indicator for the packing efficiency, is comparable to the metallic glasses. Even after temperature quench and decompression to ambient conditions, the SiO_2_-glass retains some of its unique properties at compression and possesses a Poisson’s ratio of 0.248(11). In addition to chemical alternatives in glass syntheses, coupled compression and heating treatments can be an effective means to enhance mechanical and elastic properties in high-performance glasses.

## Introduction

Glasses are indispensable materials in the industrial and technological applications owing to their unique and homogenous optical, mechanical, and chemical properties^1^. The flexibility of designing and producing a wide variety of glasses with desirable properties and chemistries have made them of considerable interests in material science and engineering applications^2^. In particular, mechanical properties, such as hardness, rigidity, and stiffness, are key to the performances of glasses and can be associated with their elastic moduli and atomic packing density (*C*_g_)^3–8^. *C*_g_, defined by the ratio between the minimum theoretical and the corresponding effective volume of glass, reflects the atomic-scale structure of glass and was recently noted to be directly correlated to the Poisson’s ratio (*ν*)^9–11^. Elastic moduli reflect the bonding strength between atoms and strongly depend on the local atomic structures. Therefore, glasses with certain elastic moduli and *ν* can thus be indicative of their potential mechanical performances.

As the archetype of widely used oxide and silicate glasses and an analogue of felsic melts in nature, SiO_2_-glass has a substantially high glass transition temperature (*T*_g_) which is an important indicator for their mechanical integrities such as hardness and stiffness in high-temperature applications^10,12–15^. Compared to the bulk metallic glasses, however, SiO_2_-glass has a significantly low *C*_g_ of 0.45 (*ν* = 0.15), bulk modulus, *K*_S_ of 33 GPa, and Young’s modulus, *E* of 70 GPa^10^. *C*_g_ and elastic moduli of SiO_2_-glass can be tailored by performing thermal treatment or chemical doping of various cations^14–17^. For example, SiO_2_-glasses doped with rare-earth oxides can have *C*_g_ and elastic moduli comparable or even greater than most of the bulk metallic glasses^18–20^. However, doping elements could lower the *T*_g_ of SiO_2_-glass^10,14–17^, which can greatly decrease the elastic moduli in the high-temperature applications^10,14–17^. The relationship between the density and thermal history of a glass can be complex^21,22^. Even with the same composition, a glass material could have different densities by varying the cooling rate and thermal history^21,22^. Designing SiO_2_- or SiO_2_-based glasses with extremely high elastic moduli by retaining a great *T*_g_ value remains to be explored^10,14–17^.

In addition to these two well-known methods, the treatment of glasses at high pressures (or compression) by shortening the interatomic distance and modifying the bonding pattern can be an effective means to induce irreversible densification and property modifications. The drawback in *T*_g_ variation in chemically-doped glasses becomes a non-factor in this process^23–30^. Since SiO_2_-glass is a key analogue to geologically abundant silicate melts, there have been a series of studies on its atomistic, elastic, and mechanical properties in compression and after high pressure–temperature (*P–T*) quenching^28–32^. Previous studies have showed that increasing pressure to 20 GPa at 300 K can lead to irreversible permanent densification of SiO_2_-glass with a gradual increase in the fraction of SiO_5_-pentahedra, SiO_6_-octahedra, and shared edges^33^. The disappearance of the SiO_4_-tetrahedra occurs between 20 and 45 GPa, accompanied by a dramatic increase in the number of SiO_6_-octahedra and shared edges and generating a more densified SiO_2_-glass^33,34^. At pressures between 45 and 140 GPa, Si–O polyhedra with a greater coordination number and connectivity will be present, producing a high-density SiO_2_-glass^28,30,33^. Simultaneous high P–T of SiO_2_-glass can be more effective in producing the aforementioned changes because the elevated temperature can help overcome the kinetic barrier of the transitions^35–43^. However, in situ characterizations of the mechanical and elastic properties of glasses at high P–T remain technically challenging. As such, hot compression of SiO_2_-glass was only performed at pressures less than 20 GPa and many previous studies focused on characterizations of quenched samples^35–43^.

**Figure 1:**
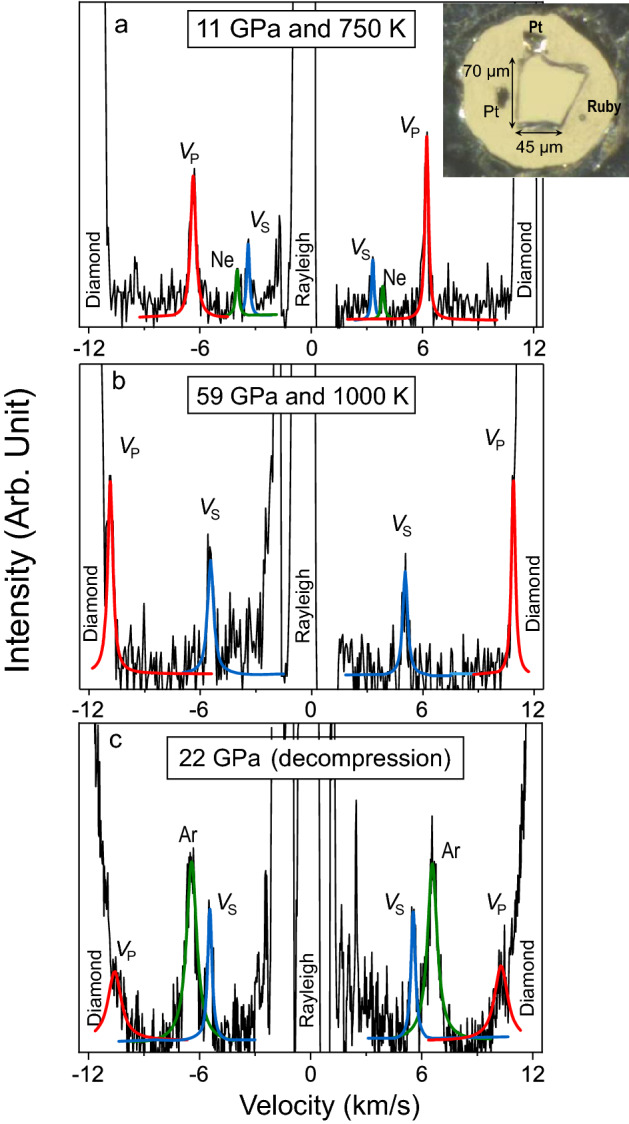
Representative Brillouin spectra of SiO_2_ glass at high pressures and temperatures. (**a**) At 11 GPa and 750 K; (**b**) at 59 GPa and 1000 K; (**c**) quenched and decompression to 22 GPa and 300 K. Black lines: raw data; red lines: fitting results for the longitudinal modes, *V*_P_; blue lines: fitting results for the transverse modes, *V*_S_. Insert figure is a representative sample photo taken at 7 GPa.

One insightful approach to evaluate mechanical and atomistic behaviours of SiO_2_-glass at high P–T is to measure its sound velocities in situ for derivations of the elastic moduli and Poisson’s ratio^10^. In this study, we have measured the compressional- (*V*_P_) and shear-wave (*V*_S_) velocities of SiO_2_-glass at simultaneously high P–T conditions up to 72 GPa at 300 K, 63 GPa at 750 K, and 59 GPa at 1000 K using Brillouin scattering in externally heated diamond anvil cells (Figs. 1 and S1, see “Methods” section for detail). The obtained results are applied to constrain *ν* and elastic moduli (*K*_S_, *G*, and *E*) simultaneously. Our results show that compression at high temperatures leads to irreversible densification and produces a dense SiO_2_-glass with ultra-high elastic moduli. More importantly, the densification process by hot compression exhibits a strong dependence on the thermal path. A dense SiO_2_-glass with ultra-high elastic moduli can be achieved at lower pressures by compression at 1000 K than that under compression at 300 K. Regardless of its thermal history, a dense SiO_2_-glass with *ν* of 0.248(11) can be retained to ambient conditions after the treatment at extreme environments. Hot compression treatment can be used as an effective means for designing dense glasses with ultra-high elastic moduli in the future.

## Results

**Figure 2:**
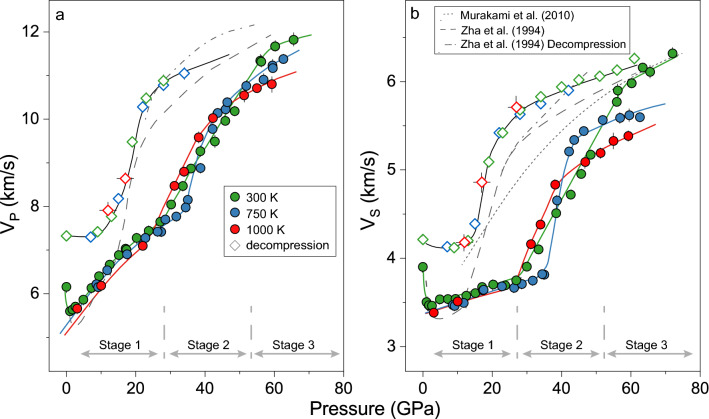
*V*_P_ and *V*_S_ of SiO_2_ glass at given temperatures. (**a**) *V*_P_ of SiO_2_ glass; (**b**) *V*_S_ of SiO_2_ glass. Green, blue, and red circles represent the velocity data at 300 K, 750 K and 1000 K by compression, respectively. Green, blue, and red diamonds are our results in decompression for SiO_2_-glass quenched from 300 K, 750 K, and 1000 K, respectively. All the colour solid lines are shown for readers to follow the trend with pressure. Grey dotted lines: SiO_2_ glass in Murakami et al. (2010)^28^; grey long-dashed lines: SiO_2_-glass in compression in Zha et al. (1994)^49^; grey dotted and dashed lines: SiO_2_-glass in decompression in Zha et al. (1994)^49^.

The *V*_P_ and *V*_S_ of SiO_2_-glass were well constrained by our Brillouin measurements up to 72 GPa at 300 K, 63 GPa at 750 K, and 59 GPa at 1000 K (Figs. 1, 2, and S1). Analysis of our velocity results show that both *V*_P_ and *V*_S_ decrease with increasing pressure up to approximately 2.5 GPa at 300 K, while they start to increase at higher pressure. The most prominent feature of the velocity–pressure profile at 300 K is the dramatic increase in the *V*_P_ and *V*_S_ between 27 and 56 GPa (Fig. 2). Above 56 GPa, the *V*_P_ and *V*_S_ of the SiO_2_-glass exhibit a nearly linear increase with pressure. These pressure-dependent features are consistent with previous high-pressure studies for the first order^26–30^.

On the other hand, high P–T experiments have revealed that sound velocities of the SiO_2_-glass have a weak dependence on temperature at pressures below 25 GPa (Fig. 2). The *V*_P_ and *V*_S_ of SiO_2_-glass at 1000 K are 3(1)% lower than those at 300 K between 1 bar and 25 GPa, respectively. At pressures between 35 and 43 GPa, the temperature effect on velocities is very different: a sudden increase in the *V*_P_ and *V*_S_ with increasing temperature was observed (Fig. 2). This anomalous change in the *V*_P_ and *V*_S_ at 750 K starts at a higher pressure but ends at a lower pressure than those at 300 K. Our velocity–pressure relation at 1000 K also exhibits an anomalous feature with a sudden increase in both *V*_P_ and *V*_S_ at 1000 K beginning at pressures as low as ~ 27 GPa and ending at 38 GPa. Interestingly, we observed a much stronger temperature effect on the reduction of the sound velocities of the SiO_2_-glass at higher pressures after the occurrence of the sudden increase: increasing the temperature from 300 to 1000 K at ~ 56 GPa leads to 6(1)% and 9(1)% reductions in the *V*_P_ and *V*_S_ of SiO_2_-glass, respectively, whereas the *V*_P_ and *V*_S_ below 25 GPa can only be lowered by 3(1)%.

After the high P–T measurements were completed at the maximum targeted pressures, electric power to the resistive heater on the diamond cell was cut off to quench the compressed SiO_2_-glass to 300 K for further decompression experiments. The temperature quenched SiO_2_-glass has *V*_P_ and *V*_S_ 39(2)% and 51(3)% larger than those at 750 K at 34 GPa, respectively (Fig. 2). More importantly, the *V*_P_ and *V*_S_ of SiO_2_-glass in decompression measurements, regardless of the previous heating temperature, were observed to consistent with each other and follow the same pressure-dependent trend within experimental uncertainties. During decompression, *V*_S_ followed a quasi-linear decrease with pressure down to 27 GPa. *V*_P_, on the other hand, was too weak to be detected during decompression until the pressure was lowered to 33 GPa. The difference in the sound velocity between decompression and compression reaches a maximum of 55(2)% at 27 GPa, showing a large hysteresis loop. We observed a sudden drop in both *V*_P_ and *V*_S_ between 27 and 13 GPa by further lowering the pressure. It is worth noting that the *V*_P_ and *V*_S_ in decompression between 1 bar and 13 GPa are 14(1)% and 15(1)% greater than those in compression at this pressure range, respectively.

## Discussion

Our high P–T sound velocity data are used to constrain the *ν* and elastic moduli of SiO_2_-glass. The obtained *V*_P_ and *V*_S_ can be directly used to calculate the Poisson’s ratio, *ν*, using the following equation:

1 $$\nu = 0.5 \times \frac{{\left( {{{V_{{\text{P}}}^{{}} } \mathord{\left/ {\vphantom {{V_{{\text{P}}}^{{}} } {V_{{\text{S}}} }}} \right. \kern-\nulldelimiterspace} {V_{{\text{S}}} }}} \right)^{2} - 2}}{{\left( {{{V_{{\text{P}}} } \mathord{\left/ {\vphantom {{V_{{\text{P}}} } {V_{{\text{S}}} }}} \right. \kern-\nulldelimiterspace} {V_{{\text{S}}} }}} \right)^{2} - 1}}$$

Together with the literature experimental equation of state results and theoretical prediction for the influence of temperature on the density of silicate melts, we have also modelled the density (*ρ*) of SiO_2_-glass at P–T conditions relevant to our measurements (see “Methods” section for details, Fig. S2). The calculated density and measured sound velocities of SiO_2_-glass were used to compute the following elastic moduli at a given P–T:

2 $$\begin{aligned} K_{{\text{S}}}^{{}} & = \rho \left( {V_{{\text{P}}}^{2} - \frac{4}{{3}}V_{{\text{S}}}^{2} } \right) \\ G & = \rho V_{{\text{S}}}^{2} \\ E & = 2G\left( {1 + \upsilon } \right) = 3K_{{\text{S}}}^{{}} \left( {1 - 2\upsilon } \right) \\ \end{aligned}$$

We estimated that varying density by 5% will cause a ± 9, ± 21, ± 23 GPa change in *K*_S_, *G*, and *E* at ~ 60 GPa between 300 and 1000 K but has no influence on the calculated *ν* (Fig. S3).

**Figure 3:**
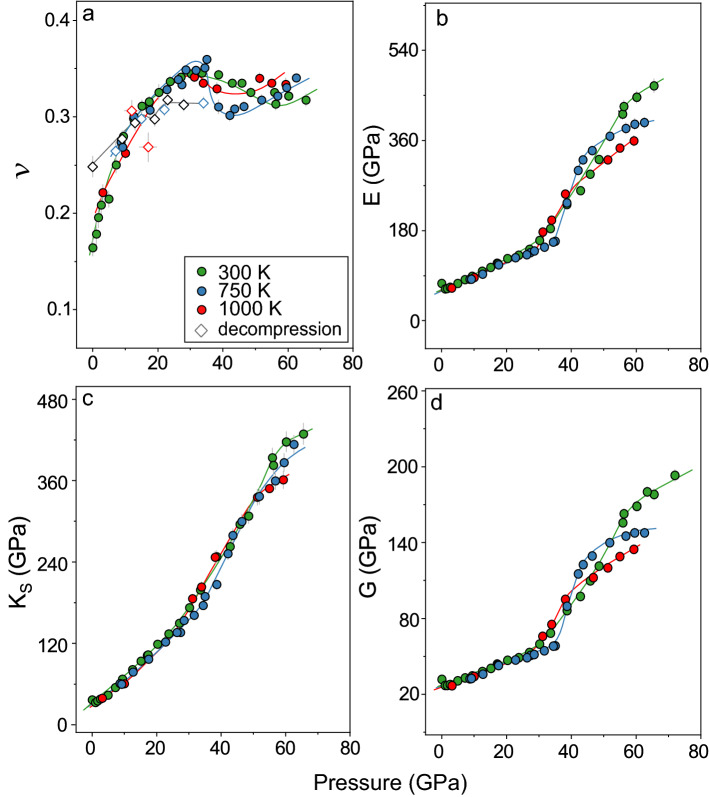
Poisson’s ratio, bulk, shear, and Young’s moduli of SiO_2_ glass at high P–T conditions. (**a**) Poisson’s ratio (*ν*); (**b**) Young’s modulus (*E*); (**c**) bulk modulus (*K*_S_); (**d**) shear modulus (*G*). Green, blue, and red circles represent the velocity data at 300 K, 750 K and 1000 K by compression, respectively. Green, blue, and red diamonds are our results in decompression for SiO_2_-glass quenched from 300 K, 750 K, and 1000 K, respectively. All the colour solid lines are shown to guides the eyes for the pressure-dependent trend.

These results reveal that elevating pressure can produce a dense SiO_2_-glass with ultra-high elastic moduli and Poisson’s ratio even at simultaneously high P–T (Fig. 3). Here the variation of elastic moduli and *ν* with pressure is discussed in three stages, which can be associated with change of atomistic behaviours of the SiO_2_-glass at high P–T conditions. The first stage occurred in our glass from ambient to 27 GPa at 300 K, which should be caused by the formation of higher-coordinated polyhedron and the transition from corner sharing to a more complex network connectivity in the SiO_2_-glass, represented by the total number of share edges per polyhedron, *n*_ES_^21,33,44^. In particular, the weak decrease in the *K*_S_, *G*, and *E* in the initial compression between 0 and 2.5 GPa should be related to the topological evolution of SiO_4_-network with an increase in entropy^45^. Further increasing pressure to 27 GPa results in a substantial increase in all the investigated elastic moduli, which are associated with the formation of SiO_5_ and SiO_6_ polyhedron and an increase in *n*_ES_ to 1.4^33^. At this pressure range, compression causes a dramatic increase in *ν*, from 0.164(8) at 1 bar to 0.341(4) at 27 GPa and 300 K, and almost double the value of *G* to 53(1) GPa and E to 143(4) GPa, while quadrupling the *K*_S_ to 150(5) GPa.

**Figure 4:**
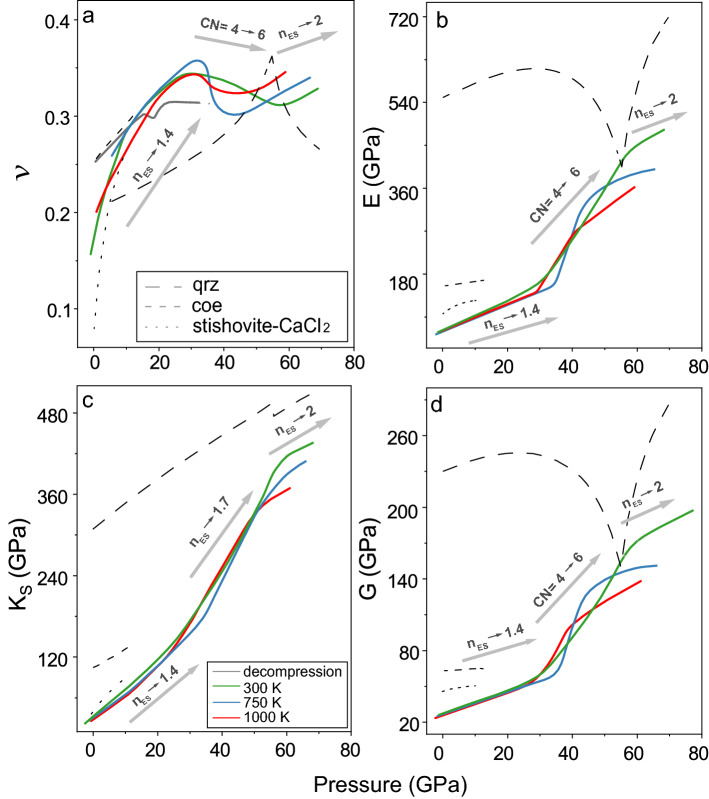
Elastic moduli of SiO_2_ glass and crystals. (**a**) Poisson’s ratio (*ν*); (**b**) Young’s modulus (*E*); (**c**) bulk modulus (*K*_S_); (**d**) shear modulus (*G*). Green lines: 300 K; blue lines: 750 K; red lines: 1000 K; grey solid line: decompression results. Grey dotted lines: quartz^51^; grey short-dashed lines: coesite^52^; grey dashed lines: stishovite and CaCl_2_-type SiO_2_^46^_._ CN: coordination number; *n*_ES_: a total number of shared edges per polyhedron. Arrows with CN on the top indicated that the CN increasing from 4 to 6 was the major reason of the moduli increasing, while the arrows with *n*_ES_ on the top indicated the moduli increasing was mostly driven by the *n*_ES_ increasing.

The sudden jump in the *G* and *E* between 27 and 56 GPa at 300 K can be associated with the second stage of the densification. At this pressure range, *K*_S_ exhibits a continuous increase with pressure from 150(5) GPa at 27 GPa to 382(14) GPa at 56 GPa and 300 K. Previous theoretical study suggested that the second stage of the densification is represented by an increase in the fraction of SiO_6_-octahedra with a jump. Meanwhile, *n*_ES_ gradually increases with pressure from 1.4 to 1.7^33^. We thus conclude that the abnormal change in the G and E between 27 and 56 GPa at 300 K should be mainly caused by the substantial increase in the number of SiO_6_-octahedra^33^. In contrast, the *K*_S_ of the SiO_2_-glass is hardly influenced by the change to a higher-coordinated structure but mainly controlled by the increase in the network connectivity. The second stage of the densification produces a SiO_2_-glass with an ultra-high *K*_S_ of 382(14) GPa, *G* of 163(4) GPa, and *E* of 428(12) GPa at 56 GPa and 300 K^33^. The *K*_S_ of this highly incompressible SiO_2_-glass is only ~ 20% lower than its crystalline counterpart, stishovite and CaCl_2_-phase SiO_2_, at 56 GPa and 300 K (Fig. 4)^46^.

Unlike the aforementioned elastic moduli, *ν* of SiO_2_-glass in the second stage of the densification exhibits a weak decrease with pressure from 0.341(4) at 27 GPa to 0.325(4) at 56 GPa (Fig. 3). This anomalous reduction in *ν* should be a result of different responses of *K*_S_ and *G* to the structural changes of SiO_2_-glass at high pressures. As noted above, the increase in *K*_S_ with pressure at 300 K is dominated by the continuous increase in the *n*_ES_, whereas the variation in *G* is more sensitive to the variation in the coordination number. The dramatic increase in *G* related to the sudden increase in the fraction of the SiO_6_-octahedra plays a major role in the observed softening of *ν* between 27 and 56 GPa at 300 K. From an atomistic perspective, the transition from the lower-coordinated polyhedron to the SiO_6_-octahedra is associated with a reduction in the ionic radius of the bonding oxygen, leading to a lower minimum theoretical volume and atomic packing density, *C*_g_^47,48^. Since *C*_g_ is directly correlated to *ν*, the dramatic increase in the SiO_6_-octahedra fraction thus produces a reduction in *ν*.

Above 56 GPa at 300 K, all the investigated elastic moduli of SiO_2_-glass follow a nearly linear increase with pressure. Most of the SiO_4_-tetrahedra and SiO_5_-pentahedra above 55 GPa have transformed into SiO_6_-octahedra^33,34^. SiO_2_-glass, dominated by the SiO_6_-octahedra, enters the third-stage of the densification. The observed increase in the investigated elastic moduli above 55 GPa should be related to the continuous increase in the *n*_ES_ to ~ 2^33^. SiO_2_-glass has a substantially great *ν* value of 0.318(6), *K*_S_ of 429(15) GPa, *G* of 178(4) GPa, and *E* of 470(13) GPa at 66 GPa and 300 K. It is interesting to note that the pressure dependence of *ν* for SiO_2_-glass in the third stage of the densification is much weaker than that below 27 GPa when the initial compression promotes the formation of higher coordination SiO_5_ and SiO_6_ polyhedron and an increase of *n*_ES_ to 1.4^33^.

We should also note that, although our measured velocities are in general agreement with previous experimental results below 12 GPa, the second stage of densification for our SiO_2_-glass occurred at a greater pressure, leading to lower *V*_P_ and *V*_S_ between 12 and 56 GPa compared to literature results (Fig. S4)^49^. The elastic moduli calculated using our velocity data in the second stage of densification are expected to be smaller than those computed using literature velocity data. The difference in the measured sound velocities between this work and previous studies could be caused by a lower initial density of 2.11 g/cm^3^ for our SiO_2_-glass^29^. This may be a result of different quench rate and thermal history when producing the SiO_2_-glass in different companies^21,44^. A higher pressure is thus required to boost the formation of SiO_6_-octahedra in the second stage of the densification for a less dense starting SiO_2_-glass. Once all the polyhedral transition into the SiO_6_-octahedra, and SiO_2_-glass enters the third stage of densification above 56 GPa, our measured sound velocities are indistinguishable from literature values. The initial state of SiO_2_-glass also influences the densification process and elastic moduli during compression.

Compared to crystalline counterparts with a similar structure, investigated elastic moduli of SiO_2_-glass exhibit a comparable temperature dependence of *G* and *E* below 25 GPa but a greater temperature dependence on *K*_S_ (Figs. 3, 4). Increasing temperature from 300 to 1000 K only lowers *K*_S_ by ~ 10(1)%, *G* by ~ 6(1)%, and *E* by ~ 6(1)% at 3–25 GPa. For comparison, *ν* of coesite (CN = 4) at 1000 K is ~ 3% lower than at 300 K, and *K*_S_, *G*, and *E* are 1%, 5% and 5% lower, respectively. More importantly, our high P–T measurements suggest that varying the temperature can lead to a completely different densification path for SiO_2_-glass. The second stage of the densification at high temperatures, represented by the sudden increase in the *G* and *E* and a decrease in *ν*, occurs between 35 and 43 GPa at 750 K and between 27 and 38 GPa at 1000 K. The transition to a structure dominated by the SiO_6_ octahedra at high temperatures occurs at a much narrow pressure range than that at 300 K, showing that a higher temperature can help SiO_2_-glass quickly overcome the energy barrier and transition to the higher coordinated structure. This also leads an abnormal temperature effect on the elastic moduli in the second stage of the densification. Between 43 and 46 GPa, *K*_S_ at 750 K becomes comparable to the value at 300 K, while *G* and *E* at 750 K are even greater. *K*_S_ at 1000 K is comparable to that at 300 K between 31 and 51 GPa, and *G* and *E* at 1000 K has a greater value. In particular, the second stage of densification at 1000 K completes at 38 GPa which is much lower than that at 300 K and 750 K. This indicates that a densified SiO_2_ glass may be more preferred at higher temperatures.

In the third stage of the densification, once most of the polyhedra transform into SiO_6_-octahedra, increasing the temperature causes a substantial decrease in the investigated elastic moduli. Temperature thus has a greater effect on the elastic moduli of densified SiO_2_-glass above 56 GPa than that with similar fractions of SiO_4_, SiO_5_, and SiO_6_ polyhedrons below 20 GPa^33^. Increasing the temperature at ~ 56 GPa from 300 to 1000 K leads to a 10(1)% reduction in *K*_S_, 18(1)% reduction in *G*, and 18(1)% reduction in *E*. The temperature effect on the *K*_S_, *G*, and *E* of SiO_2_-glass occupied by the SiO_6_ octahedra is even greater than that of its crystalline counterpart, stishovite. *K*_S_ of stishovite at 1000 K is only 10% lower than at 300 K, while *G* and *E* are 5% and 6% lower, respectively. In contrast, there is an anomalous increase in *ν* with increasing temperature above 56 GPa. Elevating the temperature from 300 to 1000 K at ~ 60 GPa can increase the value of *ν* from 0.322(4) to ~ 0.334(6).

Owing to the lack of density information during decompression, elastic moduli of SiO_2_-glass cannot be constrained after temperature quench. We thus only track the variation of *V*_P_, *V*_S_, and *ν* during decompression to the ambient conditions (Figs. 2, 3). By gradually lowering the pressure, a sudden drop in both *V*_P_ and *V*_S_ was observed between 27 and 13 GPa, which should be related to a fast increase in the fraction of SiO_4_-tetrahedra and SiO_5_-pentahedra during decompression. This structure transition has a weak effect on *ν*, represented by a weak softening. We speculate that, although most of the SiO_6_-octahedra have transitioned back to the SiO_4_-tetrahedra and SiO_5_-pentahedra below 13 GPa, the interpolyhedra connectivity dominated by edge sharing may be largely retained during decompression, leading to a more densified SiO_2_-glass with a greater *V*_P_, *V*_S_, and *ν* after the pressure was quenched to 1 bar. *ν* after decompression to 1 bar has a value of 0.248(11) which is much greater than that without treatment at extreme environments. Similar irreversible permanent densification was also observed in a previous cold-compression Brillouin study^49,50^. Although hysteresis in the *V*_P_ was observed during decompression from 57.5 to 26 GPa, most of *V*_S_ during decompression was indistinguishable from that upon compression considering the experimental errors (Figs. 2 and S4). Our high P–T data are unique in a way that they not only record the structural change of SiO_2_-glass by hot compression and decompression after temperature quench but also reveal high P–T paths to achieve ultra-high elastic moduli of SiO_2_-glass.

### Implication

**Figure 5:**
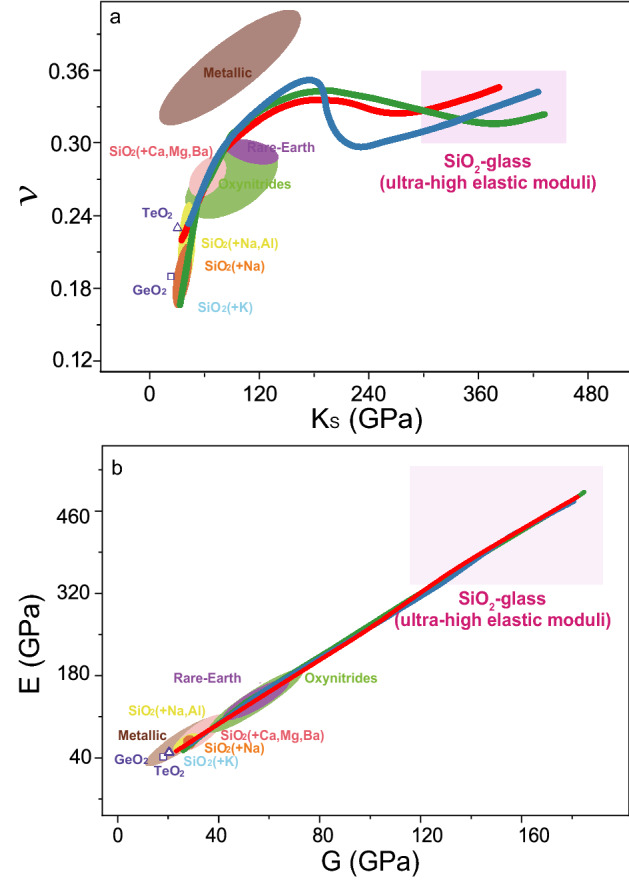
Comparison of Poisson’s ratio and elastic moduli of oxide and metallic glasses. (**a**) Poisson’s ratio (*ν*) versus bulk modulus (*K*_S_). (**b**) Young’s modulus (*E*) as a function of shear modulus (*G*). Green, blue, and red lines represent our results at 300 K, 750 K and 1000 K by compression, respectively. Triangle: TeO_2_ glass^12^; square: GeO_2_ glass^12^; light blue: SiO_2_ glass with K^16^; orange: SiO_2_ glass with Na^10^; SiO_2_ glass with Na and Al^15^; pink: SiO_2_ glass with Ca, Mg and Ba^14,17^; light green: oxynitride glasses^53^; purple: rare-Earth glasses^18,54^; brown: metallic glasses^19,55^.

We further compare the elastic moduli and *ν* of SiO_2_-glass at high P–T conditions to metallic glasses in a broad range of composition and oxide glasses enriched in SiO_2_ (Fig. 5). At ambient conditions, all the SiO_2_-based oxide glasses as well as TeO_2_ and GeO_2_ glasses have much smaller *ν* than the metallic glasses but have similar *K*_S_, *G*, and *E* values. In contrast, high P–T treatment of SiO_2_-glass can cause permanent densification and also effectively increase the elastic moduli (Fig. 5). Here, our results have shown that great *ν*, *K*_S_, *G*, and *E* are achieved when the structural network connectivity, *n*_ES_, increases to 1.4 at 27–35 GPa between 300 and 1000 K accompanied by an increase in the SiO_5_ and SiO_6_ fraction (Fig. 3). At high P–T condition above ~ 15 GPa along constant experimental temperatures in the study, SiO_2_-glass has *ν* exceeding all the SiO_2_-based oxide glasses and comparable to some of the metallic glasses. The elastic moduli, *K*_S_, *G*, and *E* are greater than the metallic glasses and comparable to rare-earth glasses and oxynitrides. Above 27–35 GPa at all the investigated temperatures, *ν*, *K*_S_, and *G* exhibit a different dependence on the structure variation in compression, but the change of *G* and *E* follow a similar trend (Fig. 5). The transition to a “stishovite-like” local structure dominated by the SiO_6_-polyhedra with an increase in *n*_ES_ to 1.7 has a minor effect on *ν* at between 27 and 56 GPa at all the investigated temperatures. The increase in *n*_ES_ during this structure transition can cause a continuous increase in *K*_S_ up to 336–394 GPa. As a result, to achieve a *ν* greater than all the metallic glasses may require high-temperature treatment of SiO_2_-glass above 72 GPa. Application of higher temperature can also help achieve a greater *ν* at a relatively lower pressure.

Unlike *ν*, the densification process at high P–T causes a dramatic increase in the elastic moduli. As mentioned above, the increase of *K*_S_ in compression at all the investigated temperatures for SiO_2_-glass is mainly controlled by the increase in *n*_ES_, while the change in *G* and *E* is dominated by the increase in the fraction of SiO_6_-octahedra (Fig. 5). After the SiO_2_-glass transitions to the “stishovite-like” local structure with *n*_ES_ ≈ 2, its *K*_S_, *G*, and *E* values have been as large as 361(9) GPa, 135(3) GPa, and 360(8) GPa at 59 GPa and 1000 K, respectively. These elastic moduli of SiO_2_-glass at 59 GPa and 1000 K are two to four times greater than those of the metallic glasses and other oxide glasses. For comparison, GeO_2_-glass follows a similar structure transition to SiO_2_-glass up to ~ 11 GPa at 300 K (Fig. S5). Yet GeO_2_-glass dominantly by the GeO_6_-octahedra only has a bulk modulus of 256 GPa at 60 GPa and 300 K, which is much smaller than the *K*_S_ of 417(14) GPa for SiO_2_-glass at the same P–T condition. Treatment of SiO_2_-glass at extreme environments can thus generate a permanently densified glass which is highly hard, rigid, and stiff. Even after the high P–T quench to ambient conditions, the densified SiO_2_-glass can partially retain a high network connectivity and has a *ν* value of 0.25, which is 55% greater than that of SiO_2_-glass counterpart at ambient and SiO_2_-glass doped Na, Al, and K as the additive^10,12–15^.

In summary, we have measured the compressional- (*V*_P_) and shear-wave (*V*_S_) velocities of SiO_2_-glass at simultaneously high P–T conditions. Hot-compression to 59 GPa and 1000 K produced a SiO_2_-glass with an ultra-high elasticity, and the structure of SiO_2_-glass during compression highly relies on the thermal path. At 59 GPa and 1000 K, SiO_2_-glass has *K*_S_ 10 times greater than that at ambient conditions, while its *G* is 5 time greater. Additionally, the Poisson’s ratio, *ν* which is an indicator for the packing efficiency, of the silica glass at 59 GPa and 1000 K is comparable to the metallic glasses. On the other hand, this dense SiO_2_-glass with ultra-high elasticity can be partially retained after decompression to the ambient conditions. The high P–T quenched SiO_2_-glass has *ν* as great as 0.248(11). Treatment the SiO_2_-glass at extreme environments is thus an effective way to increase its elasticity and enhance its mechanical performance.

## Methods

### Experimental details

SiO_2_-glass used in the experiments was purchased from Kejing, Hefei Company in China. It was produced by burning SiCl_4_ with O_2_ at 2100 °C and then quickly quenched to 300 K. The purity of the SiO_2_-glass was confirmed by electron microprobe to be 99.97% at the Material center of the University of Science and Technology of China. Measured Raman spectra of SiO_2_-glass at ambient condition were consistent with previous experimental results (Fig. S6)^11,23,26,56^. High P–T experiments were performed using the BX90 externally-heated diamond anvil cells (EHDACs) with 400 and 300-μm culet diamonds at the GeoSoilEnviro Center for Advanced Radiation Sources (GSECARS) of the Advanced Photon Source (APS), Argonne National Laboratory (ANL) and at the High-Pressure Mineral Physics Laboratory, University of Science and Technology of China (USTC)^57^. A piece of the SiO_2_-glass of a few hundred μm big was double-side polished into ~ 15-μm thick platelet. The polished platelet was cut into small pieces in a diameter of ~ 100 μm for 300-μm culet cells and ~ 120 μm for 400-μm culet cells. Two Pt foils were placed near the sample and separated by 90° as the pressure calibrant^58^. A ruby sphere was also loaded into the EHDAC as the pressure indicator during gas loading. Ne or Ar was loaded into the EHDAC at room temperature using the gas loading system at the University of Science and Technology of China as the pressure medium. The sound velocities were collected upon compression at a constant temperature (300 K, 750 K and 1000 K) (Figs. 1 and S1). For each EHDAC, we firstly increased the pressure to 4–5 GPa and then started heating, because heating the EHDAC at pressures lower than 4 GPa can easily lead to Ar or Ne leaking. Once we reached the target temperature, we waited for 20–30 min for the sample chamber’s pressure to become stable before we measured the pressure at this temperature. The sample was compressed up to 72 GPa at 300 K, while up to ~ 63 GPa at 750 K and 59 GPa at 1000 K. All the samples were quenched to 300 K after the high P–T measurements, and the velocities of the quenched sample were measured during decompression to 1 bar (Fig. S1).

Brillouin spectra were collected in a forward scattering geometry with an external scattering angle of 50° using a six-pass Sandercock tandem Fabry–Perot interferometer at GESECARS and 49.2° at High-Pressure Mineral Physics Laboratory, USTC (Fig. 1). The acoustic velocities (v) were calculated using the measured Brillouin frequency shift, Δυ_B_, in the following equation:

3 $$V = \frac{{\Delta \nu_{{\text{B}}} \lambda_{0} }}{{2\sin \left( {\theta /2} \right)}}$$

where *λ*_0_ is the incident laser wavelength (532 nm), and *θ* is the external scattering angle. The XRD patterns of Pt were collected both before and after Brillouin measurements at GSECARS of APS, ANL. XRD patterns of glass were also collected at 1000 K and 22 GPa to ensure the amorphous state of the sample (Fig. S8). The sample was also rotated by 90° to confirm its isotropy. The compression data at 300 K and all the decompression data were collected at the High-Pressure Mineral Physics Laboratory, USTC. Pressures of these data were determined by the Ruby fluorescence. Here, we used the Ruby pressure scale of Dewaele et al. (2004)^59^ which is most consistent with the metal pressure scale of Pt in Fei et al. (2007)^58^. The deviatoric stress inside the EHDAC at high P–T conditions was evaluated using the XRD peaks of Pt, which is less than 0.5 GPa (Fig. S7). Using Ar or Ne as the pressure medium can maintain a good quasi-hydrostatic experimental environment inside the sample chamber at high pressures and 300 K^60^. The deviatoric stress for our high-pressure and 300 K measurements was estimated to be less than 0.8 GPa up to 65 GPa.

We have also performed Raman measurements of the glass to characterize its properties at high P–T conditions. The collected Raman spectra up to 24 GPa at 300 K were in good agreement with literature results (Fig. S5)^11,23,26,56^. Below 6 GPa, the Raman spectra were characterized by a broad band between 188 and 554 cm^−1^ which turned into a sharp peak at 6.9 GPa with the wavenumber of 532 cm^−1^. The change in the Raman spectra below 7 GPa could be associated with the increase in the Si–O bonding ionicity. Above 14.8 GPa, a new peak appeared at 631 cm^−1^ as the shoulder of the main peak at 580 cm^−1^. The presence of this new peak should be caused by the change in the inter-polyhedra connectivity from purely corner-sharing to more complex connectivity. Above 25 GPa when the fraction of SiO_6_-octahedra starts to increase with a jump, Raman signal was lost. At 750 K, the variation of the Raman spectra with increasing pressure is the same as that at 300 K. We cannot detect the Raman signal of SiO_2_-glass above 22 GPa at 750 K (Fig. S9).

### Density calculation at high P–T conditions

Here, we followed the method of Petitgirard et al. (2017) to calculate the density of SiO_2_-glass below 60 GPa at 300 K using a second-order polynomial function^29^

4 $$\rho = - 0.00053802 \times P^{2} + 0.076204 \times P + 2.11$$

where *ρ* is density, and *P* is pressure. For pressures above 60 GPa, the density was calculated using a third-order Birch–Murnaghan equation of state with the isothermal bulk modulus, *K*_T0_ = 183 GPa, pressure derivative of the bulk modulus, *K*_T0_′ = 5, and density at ambient conditions, *ρ*_0_ = 3.95 g/cm^3^.

The effect of temperature on the density of SiO_2_-glass was calculated following the method in Stixrude et al. (2005)^61^:

5 $$P = P_{{{\text{T}}0}} + P_{{{\text{th}}}} = P_{{{\text{T}}0}} + \gamma /\nu C_{\upnu } \left( {T - T_{0} } \right)$$

and

6 $$\gamma /\nu C_{\upnu } \left( {T - T_{0} } \right) = \alpha K_{{\text{T}}} \left( {T - T_{0} } \right)$$

where *P*_T0_ is pressure at reference temperature *T*_0_ (300 K), *P*_th_ is the thermal pressure, *γ* is the Grüneisen parameter, *ν* is the Poisson’s ratio, *C*_ν_ is the constant molar heat capacity, *α* is the thermal expansion. Based on the curves of de Koker et al. (2009) for SiO_2_ liquid, the value of *αK*_T_ is assumed to be independent of temperature.

## Supplementary Information

Supplementary Information.

## Data Availability

Experimental data are listed in Tables S1 and S2. They can also be downloaded online (https://zenodo.org/record/6540997).
