# Supplementary material for: Hot dense silica glass with ultrahigh elastic moduli
Source: Sci Rep. 2022 Aug 17;12:13946. doi: 10.1038/s41598-022-18062-6 (PMC9385850; doi:10.1038/s41598-022-18062-6)
Supplement: Supplementary file 1 — Supplementary Information. [file 41598_2022_18062_MOESM1_ESM.docx]

Supplementary Materials for

**Hot Dense Silica Glass with Ultrahigh Elastic Moduli**

Ningyu Sun^1,2,3^, Zhu Mao^1,2,3*^, Xinyue Zhang^1^, Sergey N. Tkachev^4^, Jung-Fu Lin^5^

^1^Laboratory of Seismology and Physics of Earth's Interior, School of Earth and Space Sciences, University of Science and Technology of China, Hefei, Anhui 230026, China

^2^CAS Center for Excellence in Comparative Planetology, University of Science and Technology of China, Hefei, Anhui 230026, China

^3^Frontiers Science Center for Planetary Exploration and Emerging Technologies, University of Science and Technology of China, Hefei, Anhui, 230026, China

^4^Center for Advanced Radiation Sources, University of Chicago, Chicago, IL 60637, U.S.A.

^5^Department of Geological Sciences, Jackson School of Geosciences, The University of Texas at Austin, Austin, Texas 78712, USA

^*^corresponding author’s email: zhumao@ustc.edu.cn


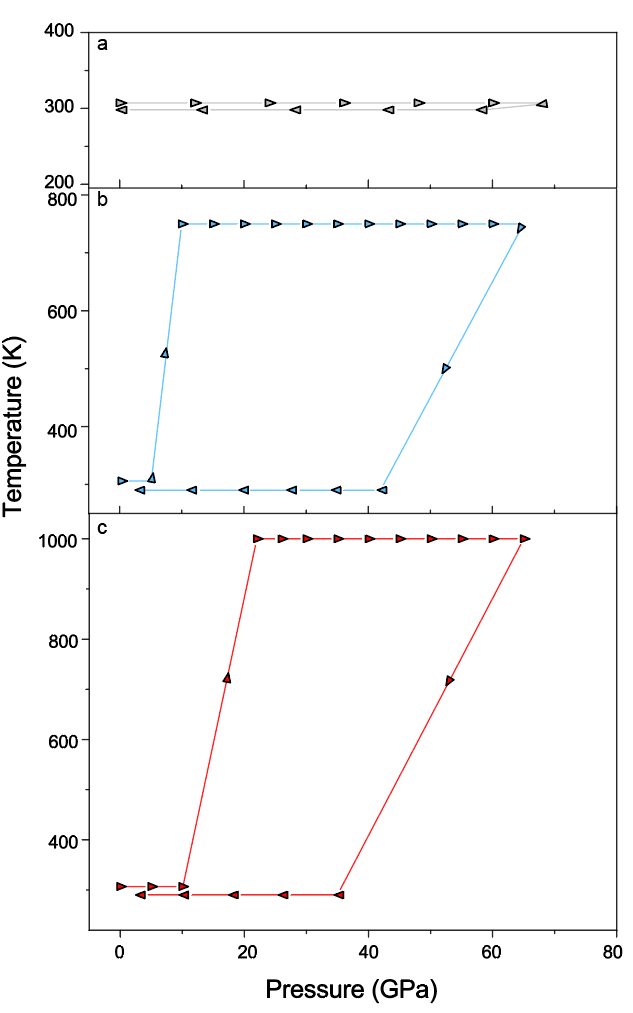


**Supplementary Figure 1.** Experimental P-T path of Brillouin measurements. (**a**) 300 K; (**b**) 750 K; (**c**) 1000 K. Arrows indicate the compression and decompression path.


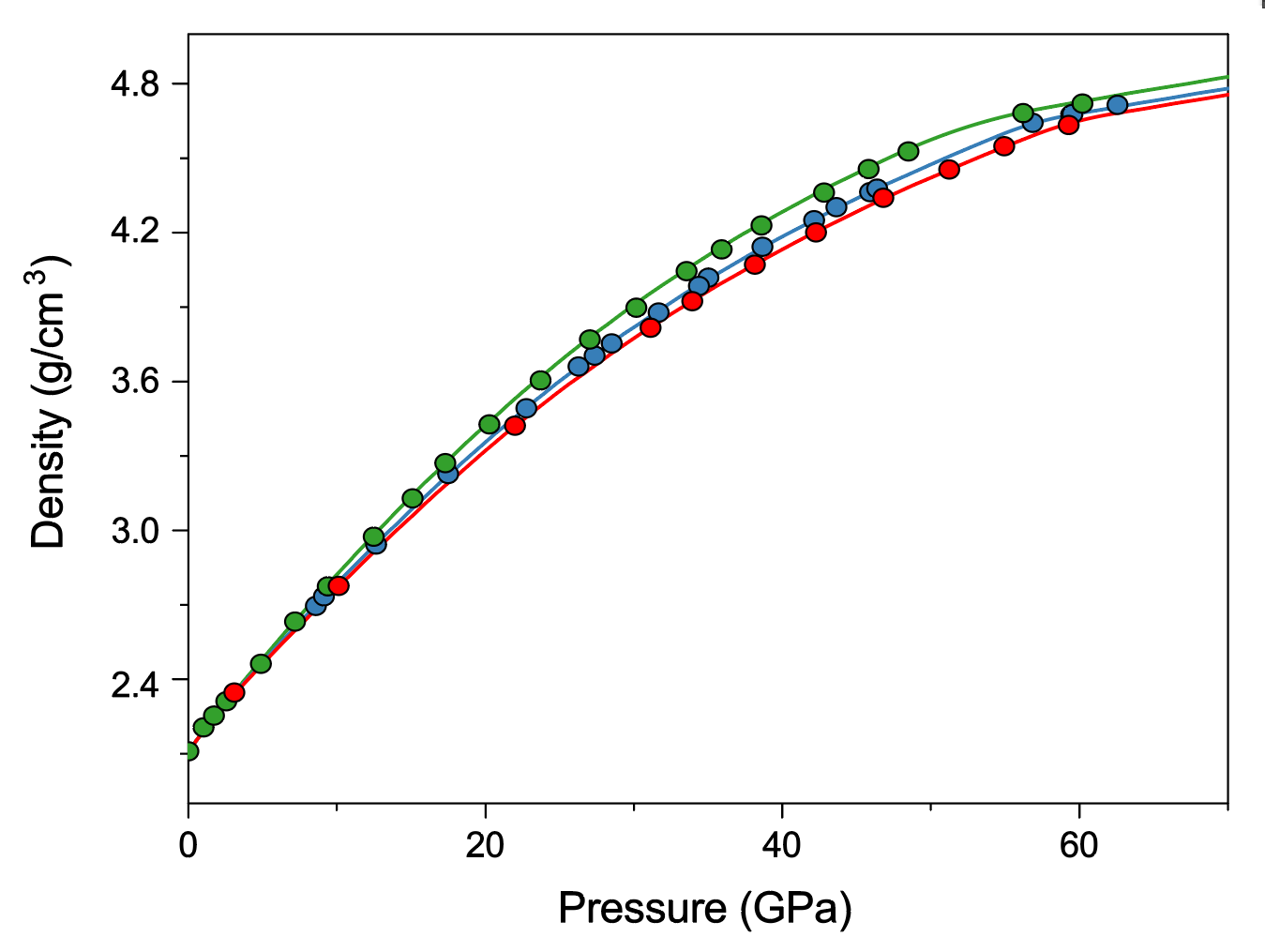


**Supplementary Figure 2.** Calculated density of SiO_2_-glass at high pressures and temperatures. Green, blue, and red circles are calculated density at 300, 750, and 1000 K, respectively. Colored lines are shown for readers to follow the trend with pressure.


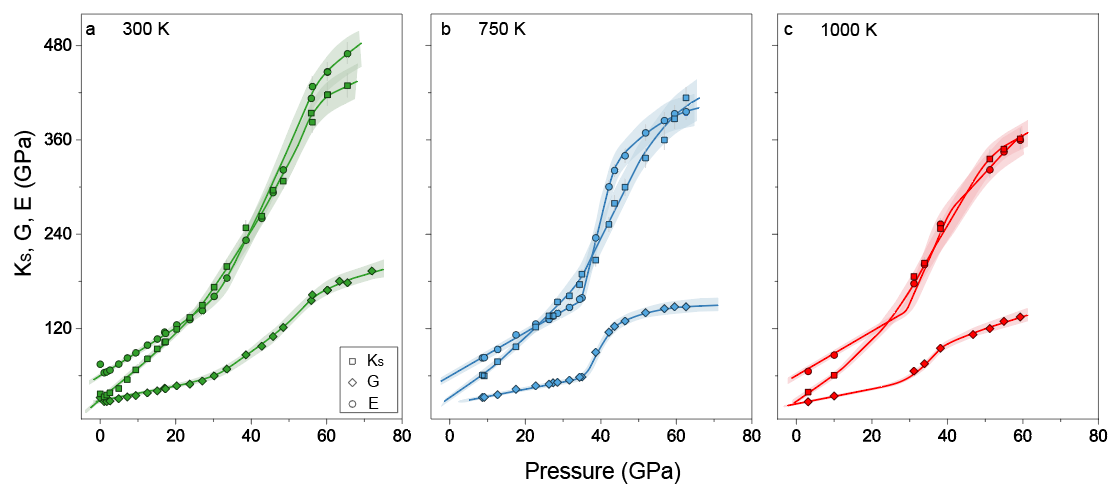


**Supplementary Figure 3.** Bulk, shear, and Young’s moduli of SiO_2_-glass at high P-T conditions. (**a**) at 300 K; (**b**) at 750 K; (**c**) at 1000 K. Circles: Young’s modulus (*E*); squares: bulk modulus (*K*_S_); circles: shear modulus (*G*). Shades indicate the uncertainties of the bulk, shear, and Young’s moduli if we vary the density by 5%.


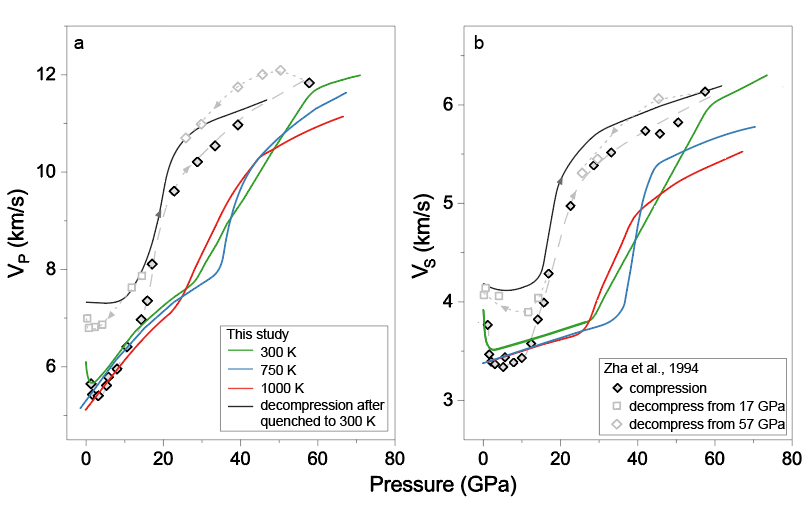


**Supplementary Figure 4.** *V*_P_ and *V*_S_ of SiO_2_-glass at high temperatures. (**a**) *V*_P_; (**b**) *V*_S_. Green, blue, and red lines represent the variation of sound velocities for our SiO_2_-glass with increasing pressure, respectively. Black lines show our decompression results. Solid diamonds and grey dashed lines: compression data in Zha et al. (1994) ^1^; open diamonds and grey dotted lines: decompression data in Zha et al. (1994) ^1^; open squares and grey dotted lines: decompression from 17 GPa in Zha et al. (1994) ^1^.


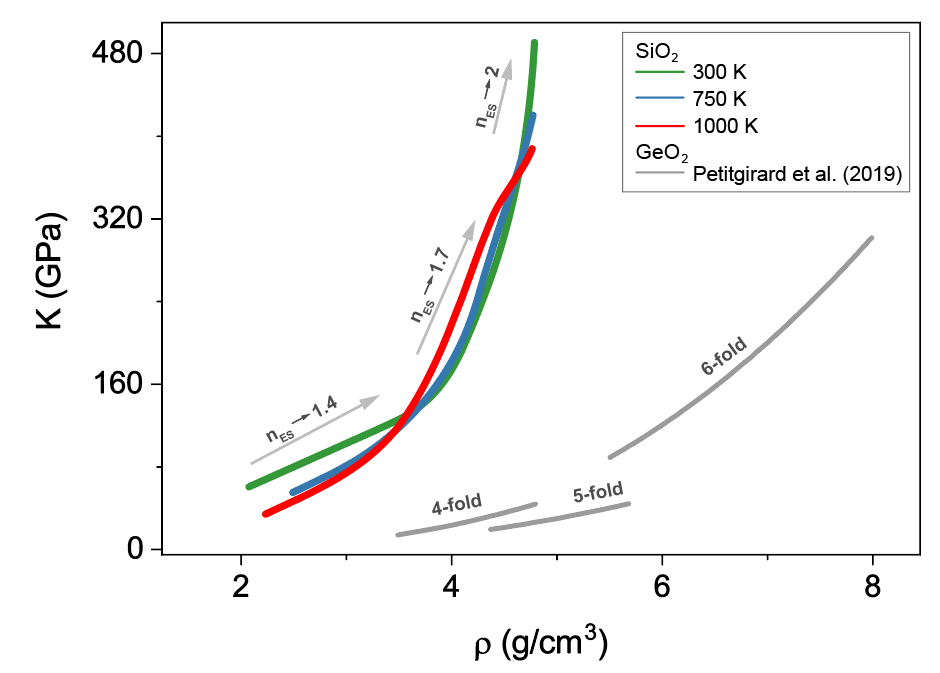


**Supplmentary Figure 5.** The variation of bulk modulus (*K*) with density (*ρ*). Green, blue, and red lines are our results of SiO_2_-glass at 300 K, 750 K and 1000 K, respectively. Grey lines are the results of GeO_2_^2^.


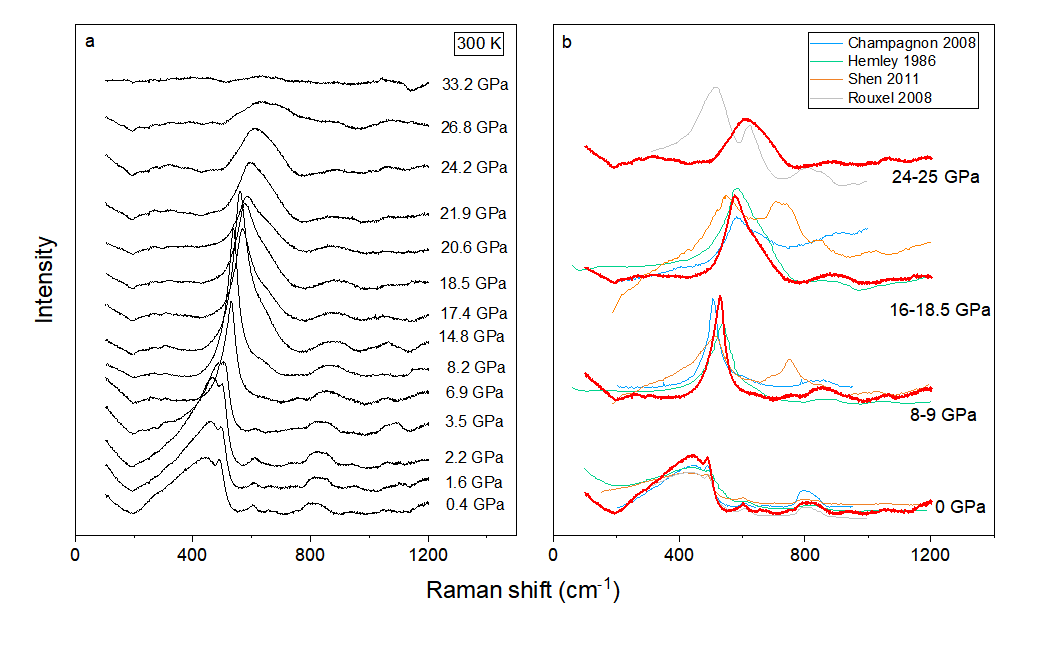
**Supplementary Figure 6.** Representative Raman spectra of SiO_2_-glass at 300 K. (**a**)Raman spectra of our SiO_2_-glass at 300 K and high pressures; (**b**) Comparison between this study and literature results. Blue lines: Champagnon et al. (2008)^3^; green lines: Hemley et al. (1986)^4^; orange lines: Shen et al. (2011)^5^; grey lines: Rouxel et al. (2008)^6^.


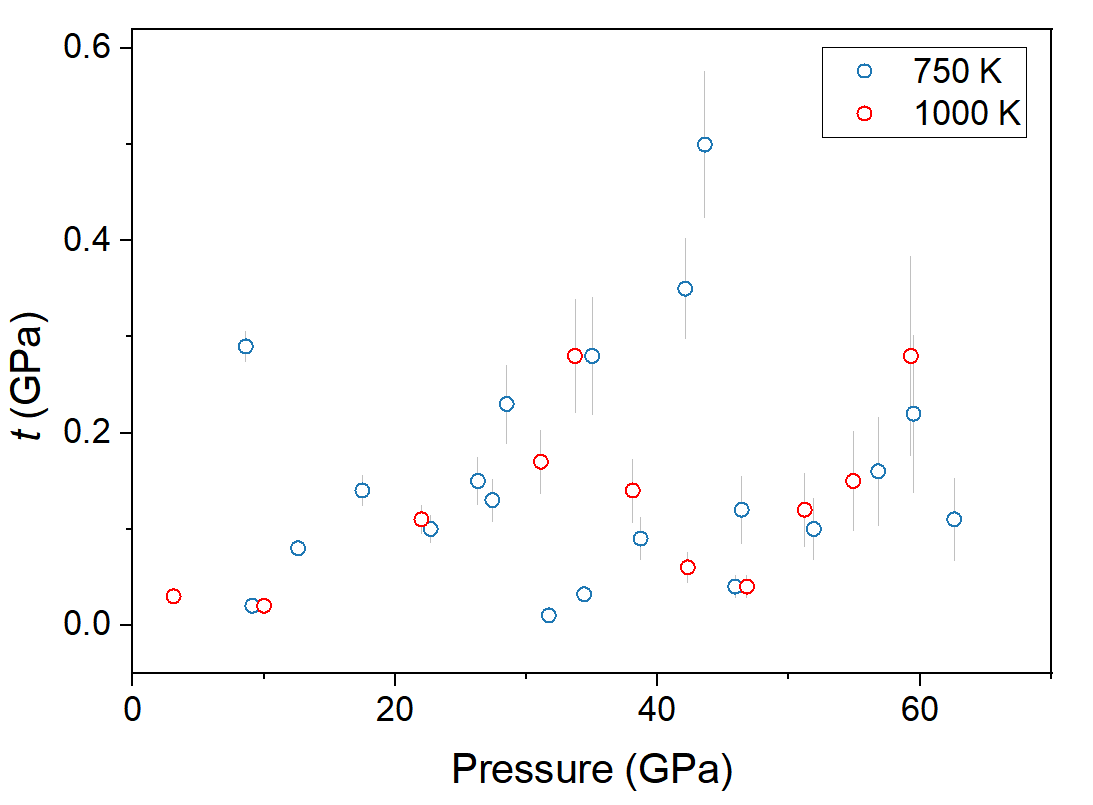


**Supplementary Figure 7.** Deviatoric stress calculated from the diffraction lines of Pt at high pressures and temperatures. Blue circles: 750 K; red: 1000 K.


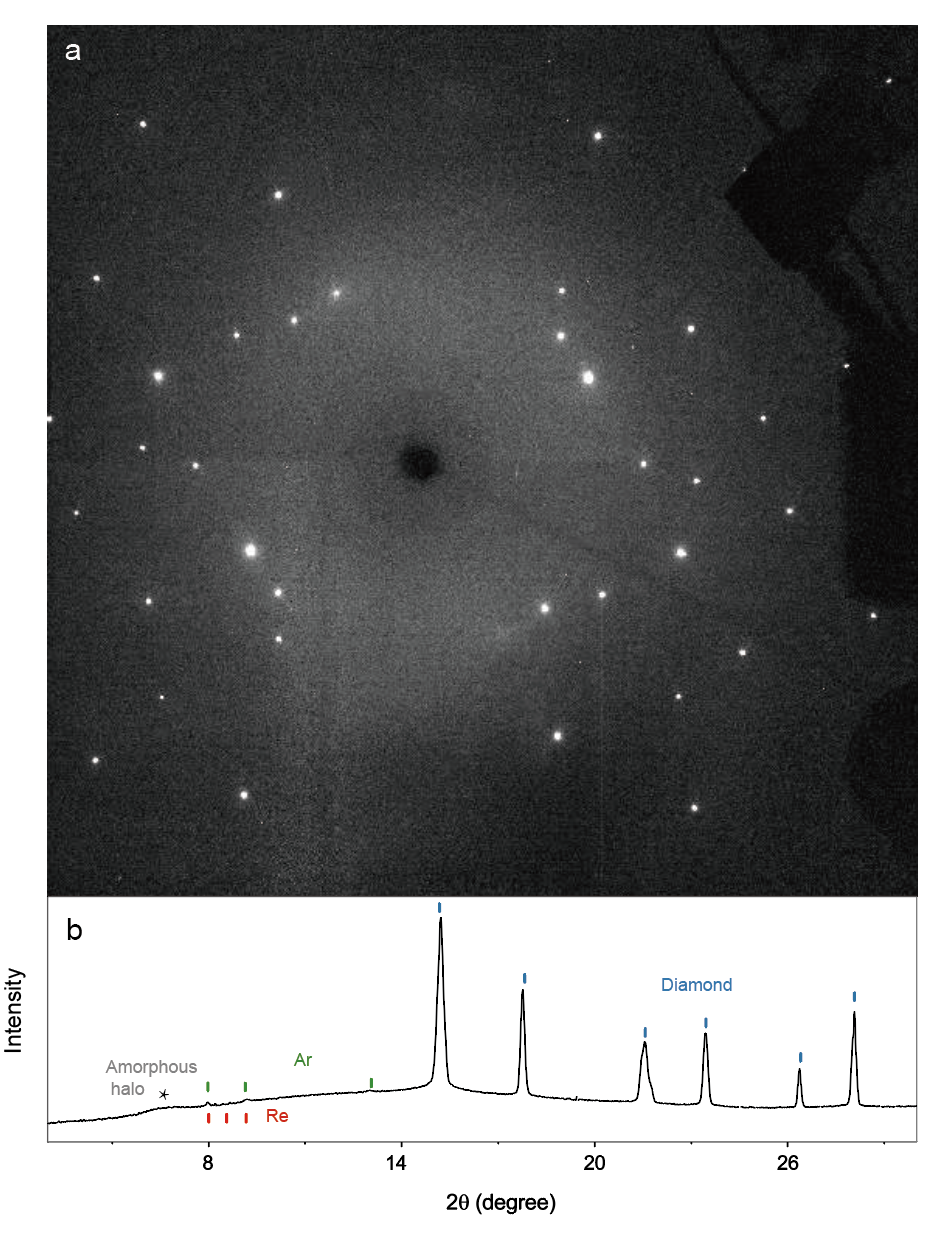


**Supplementary Figure 8.** XRD patterns of SiO_2_-glass at 22 GPa and 1000 K. (**a**) 2D diffraction patterns collected at 22 GPa and 1000 K. All the diffraction spots were from single-crystal diamonds. (**b**) Integrated 1D diffraction pattern at 22 GPa and 1000 K. Only diamond, Re (gasket material) and Ar were observed in our collected XRD patterns. Blue ticks: diamond; green ticks: Ar; red ticks: Re. Stars indicate the position of the amorphous halo.


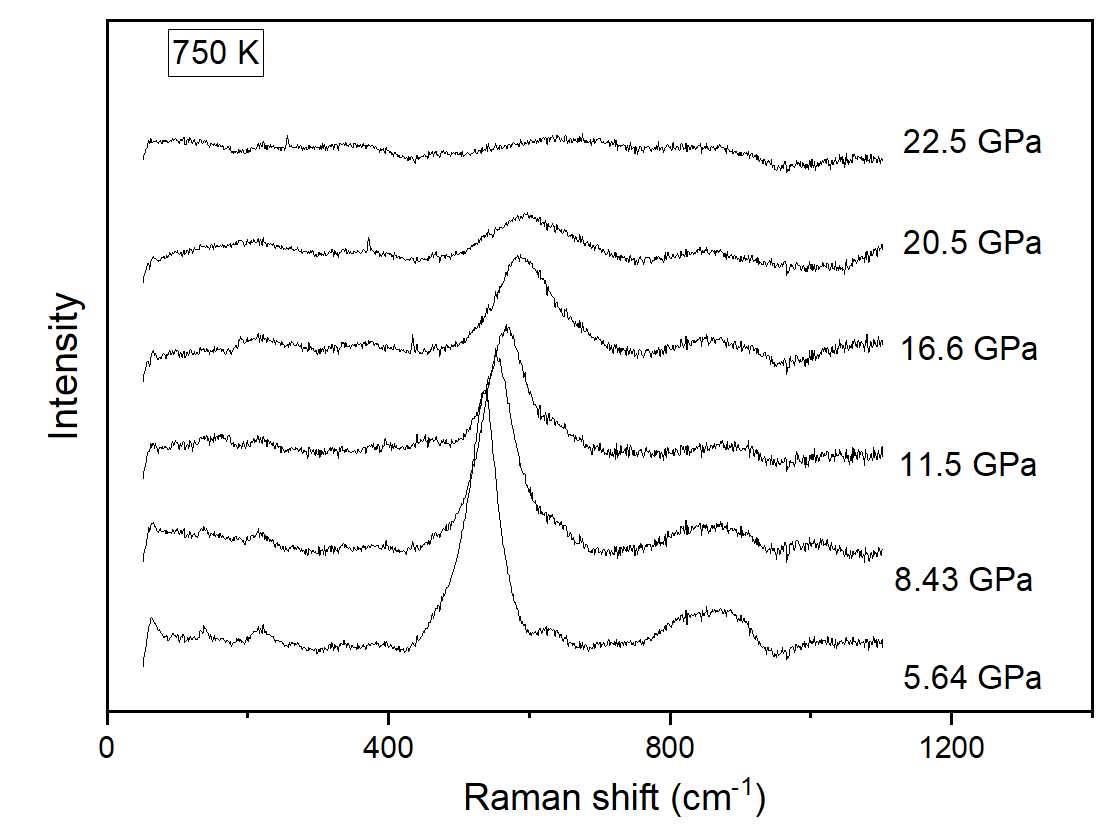


**Supplementary Figure 9.** Representative Raman spectrum of SiO_2_-glass at 750 K and high pressures.

**Table S1. Velocities and elastic parameters of SiO_2_-glass under high P-T**

| *P* (GPa) | *T* (K) | *V*_S_(km/s) | *V*_P_(km/s) | ν | ρ (g/cm^3^) | *G* (GPa) | *K* (GPa) | *E* (GPa) | *V*_Pt_ (Å^3^) |
| --- | --- | --- | --- | --- | --- | --- | --- | --- | --- |
| 0 | 300 | 3.90(2) | 6.14(4) | 0.164(8) | 2.11 | 32(1) | 37(2) | 75(2) | -- |
| 1.02(3) | 300 | 3.50(3) | 5.60(7) | 0.178(15) | 2.21 | 27(1) | 33(2) | 64(2) | -- |
| 1.72(4) | 300 | 3.47(2) | 5.64(6) | 0.196(11) | 2.25 | 27(1) | 36(2) | 65(2) | -- |
| 2.6(1) | 300 | 3.46(4) | 5.71(8) | 0.208(15) | 2.31 | 28(1) | 38(2) | 67(3) | -- |
| 4.9(1) | 300 | 3.54(2) | 5.87(5) | 0.215(8) | 2.46 | 31(1) | 44(2) | 75(2) | -- |
| 7.2(2) | 300 | 3.54(3) | 6.13(6) | 0.250(8) | 2.63 | 33(1) | 55(2) | 82(3) | -- |
| 9.4(2) | 300 | 3.54(2) | 6.41(5) | 0.280(5) | 2.77 | 35(1) | 67(3) | 89(2) | -- |
| 12.5(3) | 300 | 3.58(4) | 6.67(9) | 0.298(8) | 2.98 | 38(1) | 82(3) | 99(3) | -- |
| 15.1(4) | 300 | 3.61(3) | 6.89(10) | 0.311(6) | 3.13 | 41(1) | 94(4) | 107(3) | -- |
| 17.1(4) | 300 | 3.68(4) | 7.04(8) | 0.312(6) | 3.25 | 44(1) | 102(4) | 115(4) | -- |
| 17.3(4) | 300 | 3.64(2) | 7.01(9) | 0.316(5) | 3.27 | 43(1) | 103(4) | 114(3) | -- |
| 20.3(5) | 300 | 3.70(6) | 7.28(12) | 0.326(8) | 3.43 | 47(1) | 119(5) | 125(4) | -- |
| 23.7(6) | 300 | 3.69(3) | 7.44(13) | 0.337(6) | 3.60 | 49(1) | 134(5) | 131(4) | -- |
| 27.1(7) | 300 | 3.76(2) | 7.65(11) | 0.341(4) | 3.77 | 53(1) | 150(5) | 143(4) | -- |
| 30.2(8) | 300 | 3.92(4) | 8.05(9) | 0.345(4) | 3.90 | 60(2) | 173(6) | 161(5) | -- |
| 33.6(8) | 300 | 4.11(2) | 8.47(11) | 0.346(4) | 4.05 | 68(2) | 199(7) | 184(5) | -- |
| 35.9(9) | 300 | -- | 8.87(7) | -- | 4.13 | -- | -- | -- | -- |
| 38.6(10) | 300 | 4.52(4) | 9.27(12) | 0.344(5) | 4.23 | 86(2) | 248(9) | 232(6) | -- |
| 42.8(9) | 300 | 4.73(2) | 9.49(14) | 0.335(4) | 4.36 | 98(2) | 263(9) | 260(7) | -- |
| 45.8(11) | 300 | 4.96(3) | 9.96(9) | 0.335(3) | 4.46 | 110(2) | 296(10) | 293(7) | -- |
| 48.5(12) | 300 | 5.18(5) | 10.19(11) | 0.326(5) | 4.53 | 121(3) | 308(11) | 322(9) | -- |
| 55.9(14) | 300 | 5.77(3) | 11.34(13) | 0.325(4) | 4.68 | 156(3) | 394(14) | 413(10) | -- |
| 56.2(14) | 300 | 5.90(7) | 11.32(13) | 0.314(6) | 4.68 | 163(4) | 382(14) | 428(12) | -- |
| 60.2(15) | 300 | 5.98(4) | 11.67(13) | 0.322(4) | 4.72 | 169(4) | 417(15) | 446(12) | -- |
| 60.2(15) | 300 | 5.98(5) | 11.67(11) | 0.322(4) | 4.72 | 169(4) | 417(14) | 446(12) | -- |
| 63.4(16) | 300 | 6.16(4) | -- | -- | 4.75 | 180(4) | -- | -- | -- |
| 65.5(16) | 300 | 6.11(7) | 11.82(15) | 0.318(6) | 4.77 | 178(4) | 429(15) | 470(13) | -- |
| 72.1(18) | 300 | 6.32(6) | -- | -- | 4.84 | 193(4) | -- | -- | -- |
| 8.6(2) | 750 | 3.47(2) | 6.22(5) | 0.274(6) | 2.7 | 32(1) | 61(2) | 83(2) | 59.29(4) |
| 9.1(3) | 750 | 3.46(2) | 6.15(7) | 0.269(7) | 2.73 | 33(1) | 60(3) | 83(2) | 59.20(6) |
| 12.6(4) | 750 | 3.50(4) | 6.54(5) | 0.300(6) | 2.94 | 36(1) | 78(3) | 93(3) | 58.54(7) |
| 17.5(5) | 750 | 3.64(4) | 6.91(9) | 0.307(7) | 3.23 | 43(1) | 97(4) | 112(4) | 57.70(8) |
| 22.7(6) | 750 | 3.68(3) | 7.28(6) | 0.328(4) | 3.49 | 47(1) | 122(4) | 126(3) | 56.87(9) |
| 26.3(8) | 750 | 3.67(2) | 7.42(7) | 0.339(3) | 3.66 | 49(1) | 136(5) | 132(3) | 56.34(9) |
| 27.4(8) | 750 | 3.71(3) | 7.42(5) | 0.333(3) | 3.71 | 51(1) | 136(5) | 136(4) | 56.19(9) |
| 28.5(8) | 750 | 3.71(1) | 7.70(8) | 0.349(3) | 3.75 | 52(1) | 154(5) | 139(3) | 56.02(9) |
| 31.7(9) | 750 | 3.75(3) | 7.77(9) | 0.348(4) | 3.88 | 54(1) | 162(5) | 147(4) | 55.59(9) |
| 34.4(10) | 750 | 3.82(3) | 7.98(6) | 0.351(3) | 3.99 | 58(1) | 176(6) | 157(4) | 55.24(10) |
| 35.0(10) | 750 | 3.82(3) | 8.15(6) | 0.36(3) | 4.02 | 58(1) | 189(6) | 159(4) | 55.16(16) |
| 38.7(11) | 750 | 4.66(6) | 8.88(11) | 0.310(7) | 4.14 | 90(2) | 207(8) | 235(7) | 54.70(16) |
| 42.1(12) | 750 | 5.21(5) | 9.78(9) | 0.302(6) | 4.25 | 115(3) | 252(9) | 300(9) | 54.29(17) |
| 43.6(12) | 750 | 5.34(0) | 10.14(9) | 0.308(3) | 4.3 | 123(3) | 279(10) | 321(8) | 54.11(17) |
| 45.9(13) | 750 | -- | 10.23(10) | -- | 4.36 | -- | -- | -- | 53.85(16) |
| 46.4(13) | 750 | 5.44(4) | 10.39(10) | 0.311(5) | 4.38 | 130(3) | 300(11) | 340(9) | 53.80(17) |
| 51.9(15) | 750 | 5.57(5) | 10.76(12) | 0.317(5) | 4.52 | 140(3) | 337(12) | 369(10) | 53.20(14) |
| 56.8(16) | 750 | 5.59(6) | 10.91(13) | 0.322(6) | 4.65 | 145(3) | 360(13) | 384(11) | 52.70(15) |
| 59.5(17) | 750 | 5.62(7) | 11.17(11) | 0.331(5) | 4.71 | 148(4) | 387(13) | 393(11) | 52.43(18) |
| 62.6(18) | 750 | 5.60(5) | 11.38(10) | 0.340(4) | 2.70 | 148(3) | 413(13) | 396(10) | 52.15(21) |
| 3.1(1) | 1000 | 3.38(3) | 5.66(4) | 0.222(8) | 2.35 | 27(1) | 39(2) | 66(2) | 60.84(2) |
| 10.0(3) | 1000 | 3.51(2) | 6.19(5) | 0.262(5) | 2.78 | 34(1) | 61(2) | 86(2) | 59.38(5) |
| 22.0(6) | 1000 | -- | 7.10(6) | -- | 3.42 | -- | -- | -- | 57.23(8) |
| 31.1(9) | 1000 | 4.16(5) | 8.48(10) | 0.341(5) | 3.82 | 66(2) | 186(6) | 177(5) | 55.93(9) |
| 33.9(10) | 1000 | 4.38(4) | 8.80(11) | 0.335(5) | 3.92 | 75(2) | 203(7) | 201(6) | 55.55(15) |
| 38.1(11) | 1000 | 4.84(6) | 9.59(13) | 0.329(6) | 4.07 | 95(2) | 247(7) | 253(5) | 55.01(16) |
| 42.3(12) | 1000 | -- | 10.03(14) | -- | 4.2 | -- | -- | -- | 54.50(16) |
| 46.8(13) | 1000 | 5.09(7) | -- | -- | 4.34 | 112(3) | -- | -- | 53.97(15) |
| 51.2(15) | 1000 | 5.19(6) | 10.55(16) | 0.34(6) | 4.45 | 120(3) | 336(7) | 322(7) | 53.48(14) |
| 54.9(16) | 1000 | 5.33(9) | -- | -- | 4.55 | 129(3) | -- | -- | 53.09(21) |
| 59.3(17) | 1000 | 5.39(6) | 10.79(18) | 0.334(6) | 4.65 | 135(3) | 361(9) | 360(8) | 52.65(21) |

**Table S2. Velocities and elastic parameters of SiO_2_-glass after quench**

| *T* before quench (K) | *P* (GPa) | *V*_S_(km/s) | *V*_P_(km/s) | ν |
| --- | --- | --- | --- | --- |
| 300 | 61(2) | 6.26(7) |  |  |
|  | 56(2) | 6.13(6) |  |  |
|  | 51(2) | 6.06(5) |  |  |
|  | 45(2) | 6.02(6) |  |  |
|  | 40(1) | 5.94(6) |  |  |
|  | 34(1) | 5.83(3) |  |  |
|  | 28(1) | 5.68(5) | 10.88(14) | 0.313(6) |
|  | 23(1) | 5.42(6) | 10.48(13) | 0.317(6) |
|  | 19(1) | 5.09(5) | 9.48(12) | 0.297(7) |
|  | 13(1) | 4.20(4) | 7.77(10) | 0.294(7) |
|  | 9(1) | 4.12(4) | 7.42(9) | 0.277(8) |
|  | 0.0001 | 4.23(4) | 7.31(10) | 0.248(11) |
| 750 | 42(1) | 5.9(6) |  |  |
|  | 34(1) | 5.75(6) | 11.05(14) | 0.314(6) |
|  | 22(1) | 5.63(6) | 10.78(13) | 0.308(6) |
|  | 28(1) | 5.42(6) | 10.28(13) | 0.312(6) |
|  | 15(1) | 4.39(5) | 8.18(10) | 0.298(7) |
|  | 7(1) | 4.13(4) | 7.30(9) | 0.265(9) |
| 1000 | 27(1) | 5.71(13) |  |  |
|  | 17(1) | 4.86(11) | 8.64(14) | 0.269(15) |
|  | 12(1) | 4.18(9) | 7.96(14) | 0.306(11) |
|  |  |  |  |  |

**Reference**

1. Zha CS, Hemley RJ, Mao HK, Duffy TS, Meade C. Acoustic Velocities and Refractive-Index of SiO_2_ Glass to 57.5-GPa by Brillouin-Scattering. *Phys. Rev. B* **50**, 13105-13112 (1994).

2. Petitgirard S, Spiekermann G, Glazyrin K, Garrevoet J, Murakami M. Density of amorphous GeO_2_ to 133 GPa with possible pyritelike structure and stiffness at high pressure. *Phys. Rev. B* **100**, (2019).

3. Champagnon B*, et al.* High pressure elastic and plastic deformations of silica: In situ diamond anvil cell Raman experiments. *J. Non-Cryst. Solids.* **354**, 569-573 (2008).

4. Hemley RJ, Mao HK, Bell PM, Mysen BO. Raman-Spectroscopy of SiO_2_ Glass at High-Pressure. *Phys. Rev. Lett.* **57**, 747-750 (1986).

5. Shen GY*, et al.* Effect of helium on structure and compression behavior of SiO_2_ glass. *P. Natl. Acad. Sci. USA* **108**, 6004-6007 (2011).

6. Rouxel T, Ji H, Hammouda T, Moreac A. Poisson's ratio and the densification of glass under high pressure. *Phys. Rev. Lett.* **100**, 225501 (2008).
